# An instrument to assess the statistical intensity of medical research papers

**DOI:** 10.1371/journal.pone.0186882

**Published:** 2017-10-20

**Authors:** Pentti Nieminen, Jorma I. Virtanen, Hannu Vähänikkilä

**Affiliations:** 1 Medical Informatics and Statistics Research Group, University of Oulu, Oulu, Finland; 2 Research Unit of Oral Health Sciences, Faculty of Medicine, University of Oulu, Oulu, Finland; 3 Medical Research Center, Oulu University Hospital, Oulu, Finland; KU Leuven, BELGIUM

## Abstract

**Background:**

There is widespread evidence that statistical methods play an important role in original research articles, especially in medical research. The evaluation of statistical methods and reporting in journals suffers from a lack of standardized methods for assessing the use of statistics. The objective of this study was to develop and evaluate an instrument to assess the statistical intensity in research articles in a standardized way.

**Methods:**

A checklist-type measure scale was developed by selecting and refining items from previous reports about the statistical contents of medical journal articles and from published guidelines for statistical reporting. A total of 840 original medical research articles that were published between 2007–2015 in 16 journals were evaluated to test the scoring instrument. The total sum of all items was used to assess the intensity between sub-fields and journals. Inter-rater agreement was examined using a random sample of 40 articles. Four raters read and evaluated the selected articles using the developed instrument.

**Results:**

The scale consisted of 66 items. The total summary score adequately discriminated between research articles according to their study design characteristics. The new instrument could also discriminate between journals according to their statistical intensity. The inter-observer agreement measured by the ICC was 0.88 between all four raters. Individual item analysis showed very high agreement between the rater pairs, the percentage agreement ranged from 91.7% to 95.2%.

**Conclusions:**

A reliable and applicable instrument for evaluating the statistical intensity in research papers was developed. It is a helpful tool for comparing the statistical intensity between sub-fields and journals. The novel instrument may be applied in manuscript peer review to identify papers in need of additional statistical review.

## Introduction

All health care professionals and medical researchers face the challenge of keeping abreast of a body of knowledge that is expanding at an astonishing rate. The current views on the causes, mechanisms, and treatment methods of diseases are advancing too rapidly for any physician or researcher to achieve personal experience with all of the new findings. This has led to a growing reliance on the published literature to learn about new discoveries that can ultimately influence diagnostic evaluations, therapeutic decisions and public health guidelines.

An important function of any medical research journal is the effective dissemination of new findings to its target audience. To be an effective consumer, a journal reader should be familiar with the methodological aspects, especially when the techniques, such as statistical procedures, are invoked to clarify findings or summarize raw data. Statistical methods play an important role in medical publications. This is reflected in the high proportion of articles that are essentially statistical in character. Most papers published in medical journals contain some element of statistical methods, analysis and interpretation [1]. Statistical review has also become an important and integral part of the editorial process [2].

Because of an increasing dependence on the medical literature, it is essential to include statistical education in medical, dental and health care (undergraduate and postgraduate) training as part of the essential topics to support understanding of new research findings. Additionally, clinicians and graduated readers of medical journals should know the frequency with which various statistical concepts are reported in journals that are important to their sub-fields. This helps readers to identify the major statistical skills needed to critically evaluate their literature. Those responsible for training future practitioners and researchers to invest their resources most efficiently should ask the following questions: How often are various statistical techniques reported in the journals of a specific sub-field? Which statistical methods are mentioned most often in their journals compared to more visible journals? Do readers of clinical versus basic science journals need different statistical expertise? Has the use of statistical techniques changed over time or are there new methods that are currently applied more often?

Table 1 lists the commonly used methods to evaluate statistical significance in medical research as presented in medical statistics textbooks [3–5]. However, this table does not cover all statistical techniques used in medical, dental or health care studies. Table 2 covers other data analysis methods used for specific research questions. In some medical sub-fields or related disciplines (public health, health science, nursing, psychology), other multivariable methods such as factor analysis, structural equation models and cluster analysis are also applied. Classifying subjects or objects into predefined classes or categories is a rather common activity in sub-fields such as radiology, psychiatry or dentistry. Journals of these sub-fields often publish articles evaluating the agreement of raters using reliability coefficients. During the recent decades, mathematical statisticians have introduced new data analysis methods marked by a rapid expansion in computing capability. Examples of these are Bayesian methods, artificial neural networks (ANN) and machine learning. However, it is unclear how widely these methods are applied in different medical domains.

**Table 1:** Basic statistical methods used in medical research by research goal and type of outcome variable.

Research goal	Type of outcome variable			
	Measurement from symmetric distribution	Measurement from very skewed distribution	Categorical variable	Time to event
**Describing one variable**	Mean, SD	Median, interquartile range	Proportion	Kaplan Meier curve
**Comparing two independent groups**	Independent samples t-test	Mann-Whitney test	Chi-square test	Kaplan Meier curves and log-rank test
**Comparing three or more independent groups**	One-way ANOVA	Kruskal-Wallis test	Chi-square test	Kaplan Meier curves and log-rank test
**Comparing two repeated measurements**	t-test for repeated measurements	Wilcoxon test	McNemar test	
**Comparing three or more repeated measurements**	Repeated-measures ANOVA	Friedman test	Cochrane Q test	
**Quantifying association between two variables**	Pearson correlation	Spearman correlation	Cross-tabulation with chi-square test, RR or OR statistics	
**Explaining variation with several explanatory variables**	Multiple linear regression	Negative binomial regression	Logistic regression	Cox proportional hazard regression

**Table 2:** Advanced statistical methods.

Research goal	Brief description of methods
**Handling missing data**	Includes weighting procedures, imputation based procedures and direct model based analysis for handling incomplete data.
**Building multivariable models**	Steps for constructing a multivariable model: Stepwise variable selection, covariate adjustments, goodness of fit statistics and model validation, analyzing interaction, influence analysis and other diagnostic statistics.
**Handling repeated measurements and clustered data**	Methods for analysing clustered data where repeated measurements are made for same individuals over time or individuals are nested within groups. Extensions to basic regression methods can handle the dependencies between observations and the following terms refer to these extensions: generalized estimating equations (GEE), hierarchical models, multilevel models, nested models, generalized linear mixed models, mixed effects models, random effect models.
**Evaluating agreement**	Measures to assess agreement between raters or observers for the same set of subjects or patients. For categorical outcomes Cohen’s kappa and more stable AC1 coefficient are the most-used measures. Intra-class correlation coefficients (ICC) with several versions for different experimental designs and aims of the study are applied for assessing agreement with continuous outcomes.
**Combining results from several studies**	Meta-analysis uses data from numerous primary studies to produce an estimate of an overall associations, and explores variation between the studies.
**Reducing a dataset with many inter-correlated variables to a smaller set of variables**	Factor analysis combines multiple related variables into a small number of new variables which then represent the assumed latent characteristics in the subjects. Principal component analysis (PCA) converts a set of observations of possibly correlated variables into a set of values of linearly uncorrelated variables called principal components. PCA is mostly used as a tool in exploratory data analysis.
**Assessing unobservable latent constructs**	Structural equation models (SEM) are composed of several causal statements which hypothesize causal relationships between several observed or unobserved (latent) variables.
**Identifying groups or clusters of individuals**	Cluster analysis identifies sets of individuals who are more like each other, than they are like other individuals. This method is used to search for patterns in data and then to construct laws or rules that explain the pattern.
**Other research topics**	Bayesian methods offer an alternative way of analysing data. Bayesian statistics creates and combines numerical values of prior belief, exiting data and new data.
	Fractional polynomials, spline functions and generalized additive models (GAM) intend to extract full information from continuous variables in a multivariable setting with plausible functional form.
	Artificial neural networks and machine learning are fields of computer science that apply algorithms that can identify patterns, establish relationships to solve problems through data analysis, learn from and make predictions on these large data sets.
	Bootstrapping allows statistical inference and estimation of almost any statistic using a very general resampling procedure for estimating.
	Propensity scores are calculations of the likelihood of individuals being in a particular treatment or research group. Scores depend on those variables thought to influence group membership. Propensity score can be used as a covariate in a regression model, as a variable on which to match subjects or as a variable on which to stratify subjects.

Statistical demands are different between basic biomedical and clinical research [6]. The majority of research reports published in biomedical journals are based on animal studies with less intra-individual variation due to genetically identical species. This reduces the necessity for the application of multivariable methods to adjust for possible confounding that is typically present in clinical or epidemiological settings. The very small sample sizes associated with animal studies further lessen the possibility of applying computer-dependent statistical techniques. There are also concerns that the disregard for statistical reporting (e.g., exact sample sizes not disclosed, statistical tests used not revealed, validation of underlying assumptions not clear) in basic biomedical research articles is threatening scientific reproducibility [7,8].

A simple Medline search can reveal that many studies have examined the prevalence of different statistical methods in medical journals or groups of journals. The evidence provided dates back to the 1980s when authors were trying to identify the most frequently used statistical techniques in publications of the New England Journal of Medicine [9,10] or in major journals of some medical subfields [11–15]. This line of assessment has continued over the years to the more recent evaluations [6,16–20]. However, the authors have used different ideas for categorizations of statistical methods. In addition, most published reviews have not emphasized the quality of statistical reporting, which is an important topic for readers. Since different medical journals have distinctive requirements for the use and reporting of statistical methods and new data analysis methods are introduced, there is a need to refine the common categorizations of statistical methods by a new tool so that differences and changes in statistical intensity can be easier to evaluate and compare.

During the editorial process peer reviewers are required to comment on whether a manuscript is methodologically sound and whether the findings are clearly reported. The reviewers also need to ensure that the published manuscripts have an appropriate statistical complexity for the readers’ comprehension [21]. This approach heavily relies on the statistical expertise of subject reviewers. In general, the peer reviewers are competent in a specific range of statistical methods but they may not necessarily be aware of more general statistical issues or more recent methodological developments and best practices. Medical journals often ask their subject reviewers if they are able to assess all statistical aspects of the manuscript themselves or whether they recommend an additional statistical review [2]. Editors and reviewers may need tools (assist) for deciding when the presentation in a manuscript includes sufficient statistical methods to recommend sending the paper for a proper statistical review.

The objective of this study was to develop a reliable instrument to assess the intensity of statistical methods and reporting applicable to a wide variety of medical and health care research forums, including both clinical and basic science. In this paper, we describe this measure and findings from an initial evaluation of its reliability and functionality.

## Methods

### Statistical intensity assessment instrument

An instrument was developed to structure the assessment of the statistical intensity of an article or manuscript. Items were derived from published checklists, articles about the statistical contents of medical journal articles, editorial experience of the authors, and the comments of methodologists who reviewed the instrument drafts.

The development of the instrument originates from Emerson and Colditz [22], who classified statistical procedures into 21 categories. All these categories were included in the instrument with slight modification. The categories are used to assess the use of basic statistical methods and some specific techniques (power analysis, variable transformations, sensitivity analysis and cost-effective analysis). In the next stage, the number of items was increased by adding items that record the following information: use of p-values, confidence intervals, statistical tables and figures; description of procedures; references to statistical literature; and reporting software. This extended version, without counting the total number of different techniques used, has been applied in three bibliometric studies evaluating the use of statistical methods in psychiatry [23,24] and in dentistry [25]. Further testing of the instrument revealed that there were specialized methods or new methods that did not fit into the defined categories or items, and the instrument did not cover aspects of all statistical methods incorporated in modern medical research. After screening 840 medical papers, we included a new item for each method used in at least two articles. In addition, based on comments from medical statisticians, we also included a new group of items that measures steps related to multivariable model-building.

Testing of the instrument, review of the literature related to the topic and expert opinions resulted in the generation of 63 items pertaining to the description of statistical and data management procedures, applied statistical methods and reporting of results. Following pilot studies, the instrument was increased to 66 items. Several items were also reworded and rearranged in the evaluation form for clarity. The updated version of the instrument is included as S1 Appendix.

The instrument includes 16 groups of items. These sub-groups are denoted with capital letters (from A to P) in the evaluation form. Each group includes items measuring the usage of specific statistical analysis methods or reporting styles. Users can calculate the sums of sub-group items or a total score by summing all 66 items. In this paper, we have used the total sum of all items. We have denoted the total score as the Statistical Intensity of Medical Articles (SIMA), whose value ranges from 0 to 74. However, in practice values over 30 are very rare. A high value means that the article used several different statistical methods and reported widely varied descriptive and inferential statistics. An article with a low value for the statistical intensity means that it used few statistical methods (e.g., laboratory studies or narrative studies). A practical example of an evaluated article [26] published in the New England Journal of Medicine is given in S2 Appendix.

The statistical intensity of a published article has several dimensions, and is not simple a measure of the mathematical complexity or computer dependency of an applied method. The proposed score measures the intensity from a reader’s point of view. The instrument integrates the description of methods (section A), ancillary analysis (B, I.3 and I.4), reporting of findings using p-values, confidence intervals, tables or figures (C-E), and model-building strategies (K) with the use of a specific statistical analysis techniques. A paper with several outcomes and explanatory variables, application of multivariable methods, overuse of p-values and confidence intervals, and a very high number of tables and figures is given a high SIMA score, but medical readers might find it difficult to read.

### Set of articles

We used original research articles published between 2007–2015 in 16 journals to develop and test the scoring instrument of the statistical methods reported in medical research articles. We selected two highly visible medical journals (*Lancet and New England Journal of Medicine* (NEJM), five dental journals (*Journal of Dental Research* (JDR), *Journal of Dentistry* (JD), *Caries Research* (CR), *Community Dentistry and Oral Epidemiology* (CDOE) and *Acta Odontologica Scandinavica* (AOS)), four respiratory journals (*European Respiratory Journal (ERJ)*, *American Journal of Respiratory and Critical Care Medicine (AJRCCM)*, *Chest and Thorax)*, and five journals from other sub-fields (*Cell*, *International Journal of Epidemiology* (IJEPI), *European Journal of Public Health* (EJPH), *American Journal of Psychiatry* and *Research in Nursing and Health* (RNH) for the evaluation. We chose these journals to validate inferences about the wide range of statistical reporting in medicine, dentistry and other related fields. We analyzed a total of 240 papers published between 2007 and 2011 in the Lancet and NEJM, 200 papers published in 2010 in the five dental journals, 200 papers published in the respiratory journals during 2011 or 2015, and 200 papers published in the other journals between 2009–2013. We excluded editorials, letters, case reports and review articles from the evaluation.

### Rater reliability

The concept of inter-rater reliability has a wide range of applications across many fields of research [27]. During the conduct of a scientific investigation, classifying subjects or objects into predefined classes or categories is a rather common activity. In the proposed statistical intensity assessment instrument, research articles are classified into predefined categories of items. Most of these items have only two values (yes vs no). The reliability of this classification process can be established by asking two or more individuals referred to as raters, to independently perform this classification with the same set of articles. The extent to which these categorizations coincide represents what is often referred to as inter-rater reliability. If inter-rater reliability is high then all raters can be used interchangeably without having to worry about the evaluation of articles being affected by a significant rater factor. If interchangeability is guaranteed, then the instrument can be used with confidence without asking which rater produced them.

Four researchers (two biostatisticians and two medical researchers) with training in dentistry, epidemiology or statistics were recruited to serve as an independent panel of raters. The raters included one senior biostatistician (SB), one junior biostatistician (JB), one senior medical researcher (SMR) and one junior medical researcher (JMR). They did not receive any formal training in the use of the assessment instrument, although general guidelines were given on the instrument. The raters read and evaluated 40 randomly selected articles using the developed instrument. The articles were selected from the previously described set of 840 articles. The reliability study started in parallel with the pilot studies; thus, in their evaluation, raters used a version of the instrument that included 63 items (items B5, C5 and H2 were included after the pilot studies). The raters were not blinded to the publication journals or authors of the articles.

First, agreement between the summary score was assessed using an intra-class correlation coefficient ICC (with agreement definition, single measures, and mixed model) [28]. Generally, good agreement is defined as ICC > 0.80. To evaluate the test-retest (or intra-rater) performance the senior biostatistician read the 40 articles twice. The time interval between the scorings sessions was three years. The ICC was used to estimate the test-retest reliability.

Second, the percentage agreement, kappa coefficient and AC1 coefficient were used to assess the degree of agreement for each item [27]. The simple percentage agreement is an adequate measure of agreement for many purposes, but it does not account for agreement arising from chance alone [27,29]. Categorical agreement is often measured with Cohen’s kappa coefficient which attempts to account for the agreement that may arise from chance alone [30,31]. A kappa score in the range of 0.61 to 0.80 was considered to represent substantial agreement, and a kappa score in the range of 0.81 to 1 was considered high agreement [32]. The kappa coefficient has well-known problems when the extent of agreement between raters is high [29,33,34]. One of the problems is that a high percentage of agreement can be associated with very low kappa values, even negative values. Gwet [29] has introduced an alternative and more stable agreement coefficient referred to as the AC1 statistic.

## Results

### Statistical intensity score by the study characteristics

Table 3 shows the basic characteristics of the article set we have applied during the development of the instrument. Of the studies, 218 (26.0%) were experimental studies (clinical trials or experiments with interventions), 209 (24.9%) were observational cross-sectional studies and 142 (16.9%) were longitudinal cohort studies. The SIMA scale discriminated the articles according to their study design (p-value of ANOVA was < 0.001). Longitudinal cohort studies had the highest mean value (19.2). Meta-analyses (mean 18.5), intervention studies (17.3) and case-control studies (16.3) had values above the overall average (mean 15.4 and median 16). Case and laboratory studies that hardly apply any statistical methods, had very low score values.

**Table 3:** Basic statistics of the intensity score by study design, sample size and main outcome.

	Number of articles	Mean (SD) of SIMA score	P-value of ANOVA
**Study design**			< 0.001
• cross-sectional survey	209	15.2 (6.0)	
• longitudinal cohort study	142	19.2 (5.1)	
• case-control	49	16.3 (5.2)	
• intervention study (clinical trial)	218	17.3 (5.9)	
• reliability / diagnostic study	37	13.7 (6.4)	
• laboratory work	111	7.9 (4.3)	
• meta-analysis	39	18.5 (6.3)	
• case study	13	2.6 (2.3)	
• other	22	11.8 (7.6)	
**Sample size**			< 0.001
• <30	135	10.1 (6.0)	
• 30–99	142	13.4 (5.6)	
• 100–300	133	16.5 (5.5)	
• >300	369	19.0 (5.1)	
**Statistical significance of the main outcome**			< 0.001
• Not significant	143	17.3 (5.6)	
• Significant	486	16.9 (5.8)	
• Not evaluated	211	10.4 (7.1)	
**All**	840	15.3 (6.8)	

The mean value of the SIMA score increased with the sample size (p-value of ANOVA < 0.001). Studies with very large sample sizes (> 300) had very high scores (mean 19.0, SD 5.1). The index also identified studies that did not evaluate the statistical significance of the main outcome (Table 3).

### Statistical intensity score by the publication journal

**Fig 1:**
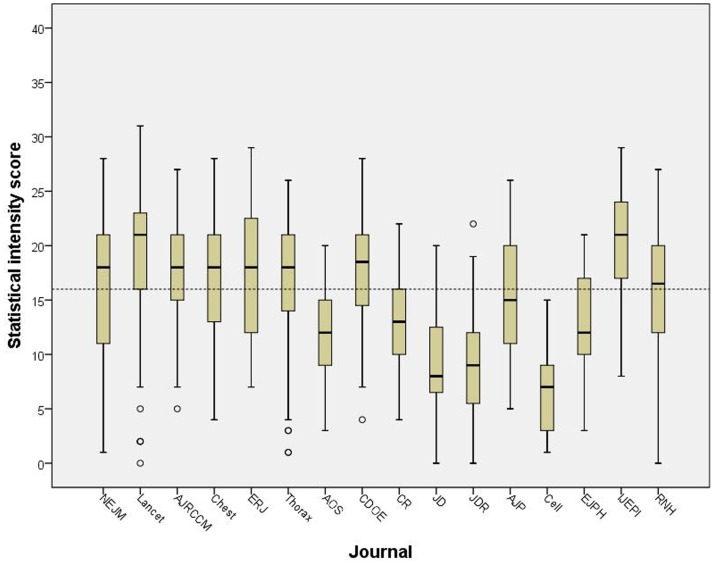
Intensity of statistical methods and reporting by the publication journal. The dotted horizontal line shows the median value of all evaluated 840 original research articles.

We also compared the statistical intensity by the publication journals. The distribution of the intensity score is shown in Fig 1. The statistical intensity was high in the very visible general medical journals (Lancet and NEJM), epidemiological journals (CDOE and IJEPI) and high impact respiratory journals. The intensity score also identified the journals (Cell, JD, and JDR) that mainly publish laboratory studies. Articles published in these journals had low scores.

### Statistical intensity score by publication year

**Fig 2:**
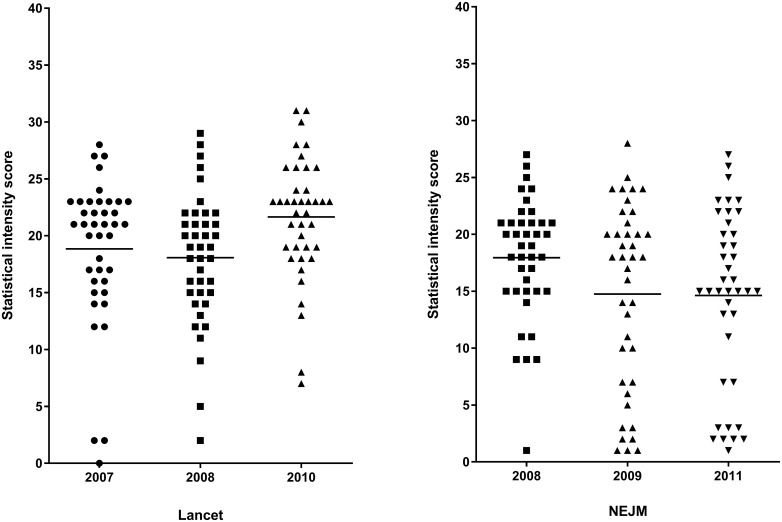
The distribution of the intensity score by publication year in the Lancet and NEJM.

To examine the sensitivity of the instrument for detecting possible changes in the statistical methods and reporting in a journal, we compared the distribution of the intensity score between different publication years in the Lancet and NEJM. The distributions are graphically presented in Fig 2. Our set of articles included a total of 120 articles published in the Lancet in 2007, 2008 or 2010. Articles published in 2010 had higher scores than articles published in 2007 or 2008 (p-value of ANOVA = 0.018). In the NEJM, articles published in 2009 or 2011 included more studies with low statistical intensity compared to articles published in 2008 (p-value of ANOVA < 0.001).

### Inter-rater and test-retest reliability

The inter-observer agreement measured by the ICC was 0.88 among all four raters. The ICC values ranged from 0.80 to 0.99 between the rater pairs (Table 4). The test-retest reliability was excellent with identical mean scores for the first and second evaluations (Pearson’s correlation coefficient was 0.99, and the intra-class correlation coefficient was 0.98).

**Table 4:** Inter-observer reliability of the statistical intensity score (SIMA score). All raters ICC = 0.878 (agreement definition, single measures, mixed model.

	**Senior biostatistician**	**Junior biostatistician**	**Senior medical researcher**	**Junior medical researcher**
**Senior biostatistician**	0.984^a^	0.943	0.909	0.843
**Junior biostatistician**			0.917	0.795
**Senior medical researcher**				0.861

^a^ Test-retest reliability

We also analyzed the inter-rater reliability between all reviewer pairs and the intra-rater reliability of one rater for each item. Because the evaluated tools included a total of 63 items, we report summary statistics of the observed reliability values by rater pairs (Table 5). The overall mean of the percentage agreement ranged from 91.7% to 95.2% and median from 95.0% to 97.5%. The intra evaluation of the senior biostatistician produced almost complete agreement (mean = 98.9% and median = 100%). Individual item analysis showed very high agreement (mean 97–100%) for the reporting of p-values or confidence intervals in abstracts and software in the methods section, the not so widely used multivariable methods (Poisson regression, SEM analysis, and cluster analysis), very widely used regression methods (logistic and Cox regression), generally adopted new methods (GEE method, meta-analysis, and methods for diagnostic tests) and some very rarely used techniques (Bayesian and machine learning methods, simulations, bootstrap or jack-knife estimations, and coast-effectives analyses). The most common disagreement among the six pairs of independent raters was in the description of statistical methods. For item A.1: “Statistical methods were described with enough detail,” the mean agreement was 55.4%. For item A.2: “Extended description of some specific procedures,” it was 66.3%. Disagreement also arose for item B.3: “Variable transforms, recodes or constructs” (mean agreement was 69.2%), item K.41: “Methods for analysing interaction” (mean agreement 75.4%) and item F.2: “Methods for proportions and cross-tabulations” (mean agreement 79.6%). Substantial agreement was achieved for the remaining items (percentage agreement was higher than 80% and AC1 coefficient higher than 0.70).

**Table 5:** Summary statistics of inter-rater (and intra-rater) reliability between the raters, based on percent agreement, kappa and AC1from a total of 63 items.

	Mean	Median	Minimum	Maximum
**SB**^a^ **vs SB re**				
• % Agreement	98.9	100.0	92.5	100.0
• Kappa	0.94	1.00	0.00	1.00
• AC1	0.98	1.00	0.90	1.00
**SB vs JB**				
• % Agreement	95.2	97.5	72.5	100.0
• Kappa	0.75	0.84	-0.03	1.00
• AC1	0.93	0.96	0.50	1.00
**SB vs SMR**				
• % Agreement	92.1	95.0	62.5	100.0
• Kappa	0.56	0.68	-0.08	1.00
• AC1	0.88	0.94	0.286	1.00
**SB vs JMR**				
• % Agreement	91.8	95.0	22.5	100.0
• Kappa	0.63	0.78	-0.04	1.00
• AC1	0.87	0.94	-0.50	1.00
**JB vs SMR**				
• % Agreement	92,9	95,0	67.5	100,0
• Kappa	0.59	0.73	-0.06	1,00
• AC1	0,86	0,95	0,41	1,00
**JB vs JMR**				
• % Agreement	91.7	95.0	25.0	100.0
• Kappa	0.62	0.77	-0.05	1.00
• AC1	0.87	0.95	-0.41	1.00
**SMR vs JMR**				
• % Agreement	92,2	95,0	40,0	100,0
• Kappa	0,57	0,70	-0,06	1,00
• AC1	0,88	0,95	-0,07	1,00

^a^ SB = senior biostatistician, SB re = senior biostatistician rescoring, JB = junior biostatistician, SMR = senior medical researcher and JMR = junior medical researcher

The mean values of the kappa coefficients ranged from 0.56 to 95.2 and median values from 0.68 to 0.84. The AC1 statistic provided high reliability values; the mean value ranged from 0.86 to 0.93 and median from 0.94 to 0.96. The mean test-retest kappa coefficient was 0.94, and the median kappa coefficient was 1, while the mean AC1 value ranged from 0.98 to 1.00.

## Discussion

Our purpose was to help readers, authors, reviewers and editors to evaluate the statistical intensity of medical research papers. Therefore, we set out with the goal of developing a new instrument for assessing the statistical methods and reporting of medical research articles, by building on empirical data from previously developed surveys, empirical evaluations and expert opinions. The composed scale measured statistical characteristics in the reviewed articles. A high value indicated that the article used several different statistical methods. The items included in the index also gave detailed information about the use of specific statistical techniques applied in the evaluated articles. The instrument had high inter-rater and intra-rater reliability.

Bibliometric data can be used to investigate the spectrum and frequency of the use of statistical techniques in medical journals. Commentaries on the use of statistical methods in medical journals reference several studies that have performed a comprehensive study of medical journals to determine the statistical methods that are most frequently used [6,35–37]. Most of these statistical content analyses have examined one specialist medical journal or general visible medical journals. There is strong evidence that simple methods such as t-tests and chi-square tests are the most common statistical techniques. The last 20 years have seen a further increase in the use of regression methods beyond these basic methods [1,19]. The availability of statistical software packages has greatly facilitated extensive data analysis, increasing the quantity and complexity of usage. Altman and Goodman [38] suggested that the following methods are likely to be seen more often in the coming years: (i) bootstrap methods, (ii) Bayesian methods, (iii) generalized additive methods, (iv) classification and regression trees (CART), (v) general estimation equations, (vi) models for hierarchical data, and (vii) neural networks. While these methods are now sometimes used in medical research, none are widely used. The instrument proposed in this paper can be applied to estimate the increased use of newer and more complex methods in different sub-fields and journals. Applying the standard evaluation form also helps to compare the findings from different fields of medicine and health care.

To evaluate the suitability of a statistical method, the authors need to ask two questions: What is our goal? What type of data have we collected?” [4]. Our first guiding principle in developing the SIMA instrument came from the observation that the use of statistical methods depends on the study design and research question. When testing the instrument on our article set, we observed that the instrument identified studies with a small sample size and research questions involving descriptive statistics. More complex longitudinal studies with large sample sizes reported a wider use of statistical analysis techniques. Our finding is in line with a previous study that notes that statistical demands are different between basic and clinical research [6]. Basic science relies on basic analyses while clinical and epidemiological studies require the application of multivariate analysis to adjust for possible confounders. The smaller sample sizes associated with animal studies further lessen the possibility of applying sophisticated statistical techniques.

Previous studies have shown that the use of statistical methods and reporting practices varies between journals, even among medical subfields [6,19,23,25,39,40]. The proposed instrument could discriminate between the journals by the statistical intensity. The visible medical journals had increased complexity of statistical analyses. In addition, the statistical intensity was higher in epidemiological journals. Statistical demands are different in basic science journals such as Cell, where Student’s t-test was often the only applied inferential statistical method. In summary, the proposed SIMA instrument can be used to review and compare the profiles of statistical content between different journals.

The proposed measure of statistical intensity was feasible. Novice raters quickly learned to use the measure with minimal training. The SIMA scale also demonstrated high inter-rater agreement and reproducibility. This high reliability among novice users supports its use by medical peer-reviewers, editors and medical educators with various expertise. We recognize the need for further testing of the SIMA assessment tool. Additional studies are necessary with a focus on the reproducibility and validity. More work is needed to improve the instrument for use in basic science research and to assess its applicability to other scientific specialties.

One of the objections raised in the evaluation process was the time and effort needed to complete 66-item evaluation checklist. It is understandable that this list seems daunting. It was our aim to develop an instrument that would be applicable to a wide variety of research types and designs. We utilized previous articles about the statistical contents of medical journal articles in developing our index. Several items were required to evaluate articles in different study designs, such as power analysis or interim analysis in clinical trials, control for confounders in observational studies, characteristics in meta-analysis or new computational methods applied in mass data analysis. Consequently, depending on the study design, only a limited number of items are applicable for each individual article. The average time of less than 20 minutes per article seemed to be a reasonable effort.

Our instrument avoids any judgement of the content, such as the originality, ethics or scientific relevance. Furthermore, we did not include items listing statistical errors. Hundreds of articles have reviewed medical papers and tried to find errors in the selection of statistical procedures [35,41–43]. The reported proportion of erroneous articles is approximately 40 to 50%. Most of the statistical problems in medical journals reported in these reviews are related to elementary statistical techniques. The errors in medical papers are probably relatively unimportant or more a matter of judgment. There is also no general agreement on what constitutes a statistical error [39,44]. We emphasize that there may be several valid ways to analyze a data set. However, we included two items (A.1 and A.2) that measured whether the reporting of the applied statistical methods was detailed, comprehensive and useful for the reader.

In some items, scoring discrepancies arose from ambiguity in the item wording. This suggests that these items were not clear for the raters. For example, in items A.1 and A.2, the junior medical researcher awarded points only when the applied methods were described with the same details as in a textbook of medical statistics. By contrast, both biostatisticians awarded a point for item A.1 when the methods section included 1–2 sentences for each basic method to describe where this method was used. In addition, they gave a point for item A.2 when the methods part of the research report included an extended description of some specific data analysis procedure. It is clear that scoring for these two items needs guidelines. We have now clarified in the instructions that the description of methods in item A.1 is incomplete if a) basic statistical methods used in the analysis were not reported in the methods section or b) only the name of a significance test given, but it was not described where the test was used. In addition, a point for item A.2 can be awarded when the following description is reported: a) model building stages or strategies in using multivariable methods, b) motivation to use an advanced or unusual statistical method, c) formulas for uncommon methods or d) background for a new methodological consideration.

Disagreement among the raters also arose when details of the study methodology appeared outside the methods section, the authors had used a method that was not described in the methods section or the methodology was not named at all. In the latter cases, raters had to make an educated guess about the potential method. The subject area knowledge and expertise were influential in scoring under these conditions and resulted in lower agreement. The limited amount of information available from the evaluated papers was the greatest challenge in the development of this tool. The use of statistical methods in a paper can be assessed to the extent that pertinent information is available in the report. In our sample, the reporting of statistical information was more detailed and comprehensive in the highly visible journals. This is probably related to consistency with their detailed guidelines for presenting statistical results as well as to a more rigorous review process, including extensive statistical reviewing [6,45]. In low-impact journals the peer review process is undoubtedly less thorough [41,46].

## Conclusions

In summary, we have developed a reliable and applicable instrument for evaluating of the statistical intensity of research papers. While most useful in the clinical and epidemiological setting, limitations may apply for the instrument’s use in basic science or non-medical fields that apply statistical methods. It may also be helpful as a checklist for preparing manuscripts or serve as an instrument for comparing the statistical intensity between journals or over time. Other possible applications include adjunct use in manuscript peer review to identify papers that require additional statistical review.

## Supporting information

S1 Appendix Statistical analyses and methods evaluation form. (PDF)

S2 Appendix A practical example of an evaluated article published in the New England Journal of Medicine. (PDF)
