# Supplementary material for: An instrument to assess the statistical intensity of medical research papers
Source: PLoS One. 2017 Oct 20;12(10):e0186882. doi: 10.1371/journal.pone.0186882 (PMC5650171; doi:10.1371/journal.pone.0186882)
Supplement: S2 Appendix — (PDF) [file pone.0186882.s002.pdf]

Statistical analyses and methods evaluation form

Article id number: 629

Reviewer: PN

Evaluated: 21.10.2016

Journal name: NEJM

Volume and pages: 365: 689-698

Year: 2011

DOI: 10.1056/NEJMoa110423

Authors

Name of the first author: ALBERT RK

Design, software and primary outcome

Study design:

1. cross-sectional survey
2. longitudinal or cohort study
3. case-control
4. intervention study (clinical trial) ✓
5. reliability / diagnostic study
6. laboratory work
7. meta-analysis
8. case study
9. other

Sample size: 1117

First software: -

Second software: -

Statistical significance of the primary outcome:

0. Not significant
1. Significant ✓
2. Not evaluated

		No	Yes		
A. Description of methods					
Item A.1 Statistical methods described with enough detail		0	1✓		
Item A.2 Extended description of some specific procedures		0✓	1		
Item A.3 Software reported		0✓	1		
Item A.4 Reference to statistical literature		0	1✓		
B. Ancillary analyses and computations					
Item B.1 Power analysis and sample calculations		0	1✓		
Item B.2 Methodological considerations		0✓	1		
Item B.3 Variable transforms, recodes or constructs		0✓	1		
Item B.4 Sensitivity or influence		0✓	1		
Item B.5 Stratification or sub-group analyses		0✓	1		
C. Reporting of statistical inference					
Item C.1 P-values		0	1✓		
Item C.2 Confidence intervals		0	1✓		
Item C.3 P-values or confidence intervals in abstract		0	1✓		
Item C.4 Adjustment of p-values (multiple comparisons)		0	1✓		
Item C.5 Actual p-values reported		0	1✓		
D. Number of p-values and CIs in tables and figures		0	1-9	10-29	≥ 30
Item D.1 Number of p-values	[N1= 12]	0	1	2✓	3
Item D.2 Number of CIs	[N2= 15]	0	1	2✓	3
E. Number of statistical tables and figure		0	1-2	3-4	≥ 5
Item E.1 Number of tables	[N3= 2]	0	1✓	2	3
Item E.2 Number of figures	[N4= 3]	0	1	2✓	3
F. Basic inferential methods for comparing groups					
Item F.1 Comparison of means		0	1		
Item F.2 Methods for proportions and cross-tabulations		0	1		
Item F.3 Non-parametric methods		0	1✓		
G. Basic methods for repeated measurements					
Item G.1 Comparison of means		0✓	1		
Item G.2 Methods for proportions and cross-tabulations		0✓	1		
Item G.3 Non-parametric methods		0✓	1		
H. Correlation coefficient methods					
Item H.1 Pearson's correlation coefficient or related		0✓	1		
Item H.2 Rank correlation coefficients		0✓	1		
I. Other basic methods					
Item I.1 Kaplan-Meir curve		0	1✓		
Item I.2 Epidemiological measures of disease frequency		0	1✓		
Item I.3 Evaluation of normality		0✓	1		
Item I.4 Missing data methods		0✓	1		
Item I.5 Comparison of variances		0✓	1		

	No	Yes
J. Regression methods		
Item J.1 Linear regression	0✓	1
Item J.2 Logistic regression	0✓	1
Item J.3 Cox regression	0	1✓
Item J.4 Poisson or negative binomial regression	0	1✓
Item J.5 Other link function	0	1
K. Multivariable model-building		
Item K.1 Assessing assumptions	0	1
Item K.2 Methods for stepwise variable selection	0	1
Item K.3 Covariate adjustments	0	1✓
Item K.4 Goodness of fit statistics and model validation	0	1
Item K.5 Methods for analyzing interaction	0	1
Item K.6 Influence analysis and other diagnostic statistics	0	1
L. Intra-cluster correlation methods		
Item L.1 GEE	0✓	1
Item L.2 Random effects models or mixed models	0✓	1
Item L.3 Complex surveys, robust estimates or other	0✓	1
M. Measures of agreement and diagnostic tests		
Item M.1 Assessing agreement with continuous outcomes	0✓	1
Item M.2 Agreement for categorical classifications	0✓	1
Item M.3 Methods for diagnostic tests	0✓	1
N. Meta-analysis		
Item N.1 Overall CI or p-value	0✓	1
Item N.2 Test of homogeneity or meta-regression	0✓	1
Item N.3 Other meta-analysis procedure	0✓	1
O. Other multivariable methods		
Item O.1 Factor analysis	0✓	1
Item O.2 Principal component analysis	0✓	1
Item O.3 SEM	0✓	1
Item O.4 Cluster analysis	0✓	1
Item O.5 Other multivariable method not classified above	0✓	1
P. Other techniques		
Item P.1 Methods for analyzing time series	0✓	1
Item P.2 Bayesian methods	0✓	1
Item P.3 Curve fitting, spline functions or GAM	0✓	1
Item P.4 Statistical genetics and microarray data	0✓	1
Item P.5 Artificial neural networks or machine learning	0✓	1
Item P.6 Simulations	0✓	1
Item P.7 Bootstrap or jack-knife estimates	0	1✓
Item P.8 Interim analyses	0	1✓
Item P.9 Cost-effectiveness analyses	0✓	1
Item P.10 Other technique not classified in earlier items	0✓	1

Total score = 23
